# Rifaximin Use, Adherence and Persistence in Patients with Hepatic Encephalopathy: A Real-World Study in the South of Italy

**DOI:** 10.3390/jcm12134515

**Published:** 2023-07-06

**Authors:** Fulvio Pandico, Anna Citarella, Simona Cammarota, Francesca Futura Bernardi, Ernesto Claar, Carmine Coppola, Marianna Cozzolino, Federica De Rosa, Massimo Di Gennaro, Marianna Fogliasecca, Roberta Giordana, Daniela Pacella, Alessandro Russo, Vito Salerno, Luca Scafa, Ugo Trama

**Affiliations:** 1 Department of Territorial Pharmaceuticals, Local Health Authority of Caserta, 81100 Caserta, Italy; fulvio.pandico@aslcaserta.it (F.P.); marianna.cozzolino@aslcaserta.it (M.C.); 2 LinkHealth Health Economics, Outcomes & Epidemiology S.R.L., 80143 Naples, Italy; simona.cammarota@linkhealth.it (S.C.); marianna.fogliasecca@linkhealth.it (M.F.); 3 Regional Pharmaceutical Unit, Campania Region, 80143 Naples, Italyugo.trama@regione.campania.it (U.T.); 4 Internal Medicine and Hepatology Unit, Ospedale Evangelico Betania, 80147 Naples, Italy; ernestoclaar@gmail.com; 5 Unit of Hepatology and Interventional Ultrasonography, Department of Internal Medicine, OORR Area Stabiese, 80054 Gragnano, Italy; c.coppola@aslnapoli3sud.it; 6 Postgraduate School in Clinical Pharmacology and Toxicology, Department of Medicine, Surgery and Dentistry “Scuola Medica Salernitana”, University of Salerno, 84081 Baronissi, Italy; fderosa@unisa.it; 7 Innovation and Data Analytics (So.Re.Sa), Campania Region, 80143 Naples, Italy; m.digennaro@soresa.it; 8 Monitoring of Public Healthcare System (So.Re.Sa), Campania Region, 80143 Naples, Italy; r.giordana@soresa.it (R.G.); a.russo@soresa.it (A.R.); v.salerno@soresa.it (V.S.); l.scafa@soresa.it (L.S.); 9 Department of Public Health, University of Naples Federico II, 80131 Naples, Italy; daniela.pacella@unina.it

**Keywords:** hepatic encephalopathy, rifaximin, adherence, persistence, drug utilization, real-world data

## Abstract

Real-world data on the therapeutic management of hepatic encephalopathy (HE) patients are limited. The aim of this study was to evaluate the HE medications prescribed in an Italian cohort of HE patients post-discharge and to assess the real-world rifaximin adherence and persistence over 1 year. An observation retrospective study was conducted using data retrieved from outpatient pharmaceutical databases and hospital discharge records of the Campania region. For all subjects hospitalized for HE during 2019 (cohort 1), the HE medications prescribed within 60 days after discharge were evaluated. Adherence (proportion of days covered, PDC) and persistence were estimated for rifaximin 550 mg incident users over 1 year (cohort 2). Patients with PDC ≥80% were considered adherents. Persistence was defined as the period of time from the first rifaximin prescription to the date of discontinuation. Discontinuation was assessed using the permissible gap method. In cohort 1, 544 patients were identified; 58.5% received rifaximin while 15.6% only received non-absorbable disaccharides and 25.9% did not receive any HE medications. In cohort 2, 650 users were selected; only 54.5% were adherents and 35% were persistent users at 1 year. This real-world study highlights that quality improvement in therapeutic management is needed to potentially improve the outcomes of HE patients.

## 1. Introduction

Hepatic encephalopathy (HE) is one of the most debilitating complications of cirrhosis and severely affects the lives of patients and their caregivers. It is characterized by reversible neurological impairments that result from the accumulation of inflammatory cytokines and elevated levels of gut-derived neurotoxins (e.g., ammonia). Symptoms start with psychomotor changes and progress to confusion, disorientation and eventually coma. Hospitalizations occur frequently to manage these debilitating symptoms. Patients with a previous history of overt HE have a 42% risk of recurrence within one year. A retrospective analysis of a large Italian cohort with cirrhosis reported that the presence of hepatic encephalopathy was the strongest predictor of hospitalization for any cause [1]. Furthermore, HE occurrence is associated with poor survival, with a 1-year survival rate of 40% to 50% and a 3-year survival rate of approximately 20% [2,3]. Frequent occurrence of HE and high hospital admission rates result in a significant economic burden on healthcare systems [4]. Notably, HE has a substantial economic effect, not only owing to the direct costs of its management but also to the indirect costs arising from the absence from work and loss of work productivity for both patients and caregivers [5,6].

Rifaximin-α 550 mg is a poorly absorbed bacteriostatic rifamycin derivative that was approved for reducing the risk of overt HE recurrence in adults by the Italian Medicines Agency in 2017. Rifaximin inhibits bacterial protein synthesis by irreversibly binding to the beta-subunit of the deoxyribonucleic acid (DNA)-dependent ribonucleic acid (RNA) polymerase enzyme in bacteria. It has a broad antimicrobial spectrum of activity against both Gram-positive and Gram-negative bacteria, especially anaerobic enteric bacteria, including ammonia-producing species. It is thought to inhibit the division of urea-deaminating bacteria, thereby reducing the intestinal production of ammonia and other compounds that are believed to be important to the pathogenesis of HE [7]. Another interesting aspect is that rifaximin-α has been shown to act as a eubiotic drug, promoting the growth of beneficial bacterial such as Lactobacilli in cirrhotic patients with HE [8]. Furthermore, rifaximin-α treatment downregulates the inflammatory response by reducing the expression of proinflammatory cytokines tumour necrosis factor-alpha and interleukin-1, inhibiting the activation of nuclearfactor kappa B and reducing bacterial translocation and virulence without significantly changing the overall composition of the gut microbiota [9,10].The addition of rifaximin to non-absorbable disaccharides has been found to be superior to both treatment with non-absorbable disaccharides or rifaximin alone in terms of having an increased effective rate and decreased mortality in patients with HE [11,12,13,14,15]. In a 24-month open-label maintenance study, rifaximin-α appeared to provide a continued reduction in HE-related and all-cause hospitalization without an increase in adverse events [16]. The European Association for the Study of the Liver (EASL) guidelines recommend the use of rifaximin in combination with lactulose as secondary prophylaxis of overt HE recurrence. Despite the evidence-based effectiveness of rifaximin, implementation gaps exist that are frequently related to prescribing behaviours. Moreover, adherence to medications is essential to prevent the occurrence of precipitating factors for HE events, such as constipation and infection [17,18]. However, data to inform real-world patient persistence and adherence to rifaximin are currently limited.

On the basis of these considerations, the aim of this study was to evaluate the prescribing of rifaximin in a large Italian cohort of patients after a hospitalization for an HE event and to assess the real-world adherence to and persistence with rifaximin over a 1-year period.

## 2. Methods

### 2.1. Data Sources

This retrospective observational study was conducted using data retrieved from outpatient pharmaceutical databases and hospital discharge records (HDRs) of the Campania region in the south of Italy (accounting for almost 10% of the Italian population). Briefly, outpatient pharmaceutical databases include dispensing date and anatomical therapeutic chemical (ATC) codes of drugs prescribed by specialists and primary care physicians and reimbursed by the Italian National Health Service (INHS); HDRs collect information on patients’ hospital discharge date and discharge diagnosis and up to 5 secondary diagnoses and procedures recorded according to the International Classification of Diseases, Ninth Revision, Clinical Modification (ICD-9-CM). All data were linked through the unique individual identification code, which was properly anonymized to respect the subject’s privacy. To guarantee the patients’ privacy, each identification code was automatically anonymized, in full compliance with the European General Data Protection Regulation (GDPR) (2016/679), and the inverse process is only allowed if permission is granted to the regional authority upon request from judicial authorities.

### 2.2. Study Design and Cohort Selection

#### 2.2.1. Cohort 1—HE Hospitalized Patients

**Figure 1:**
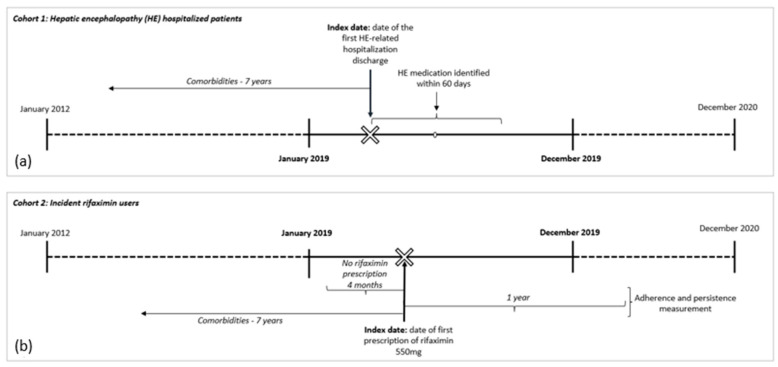
Study design: (**a**) cohort 1— HE hospitalized patients; (**b**) cohort 2—Incident rifaximin users.

For the first aim, all residents in the Campania region, 18 years and older, who were hospitalized with a diagnosis code for HE (ICD9-CM 572.2) between 1 January 2019 and 31 December 2019 (enrollment period) were identified in the HDRs (Figure 1a). The date of the first hospitalization during the enrollment period was defined as the index date.

#### 2.2.2. Cohort 2—Incident Rifaximin Users

For the second aim, all residents in the Campania region, 18 years and older, receiving rifaximin 550 mg (ATC code A07AA11) between 1 January 2019 and 31 December 2019 (enrollment period) were identified in the outpatient pharmaceutical databases (Figure 1b). In Italy, rifaximin at a dosage of 550 mg twice daily is specifically authorized and reimbursed only for reducing the risk of overt HE recurrence in patients 18 years of age or older. The date of the first prescription during the enrollment period was defined as the index date of the follow-up. Patients who received a rifaximin prescription during the 120 days before the index date were excluded.

#### 2.2.3. Outcome for Cohort 1

For each subject in cohort 1, we evaluated the prescribing of medication within the first 60 days after discharge for HE and classified it as follows: the rifaximin group included subjects receiving at least one prescription of rifaximin (ATC A07AA11) with or without non-absorbable disaccharides; the non-absorbable disaccharides group, those receiving lactulose (ATC A06AD11) or lactitol prescriptions (ATC A06AD12) alone; the no-treatment group, those with no prescription of rifaximin or non-absorbable disaccharides.

#### 2.2.4. Outcome for Cohort 2

For each subject in cohort 2, we estimated the adherence to and persistence with rifaximin. Adherence was estimated as proportion of days covered (PDC) over the year following the first prescription of rifaximin (index date) or until death, whichever occurred the first. The PDC was calculated as the total number of medication-covered days divided by the number days in the follow-up period. The rifaximin daily dosage was assumed to be 1100 mg as recommended by the guidelines [18,19,20]. PDC was dichotomized and patients with a PDC value ≥80% were considered adherents [21].

Persistence with rifaximin use was defined as the period of time from the index date to the date of discontinuation [22]. Discontinuation was assessed using the permissible gap method [23]. Gaps are periods in which no medication is available to the patient, and treatment was considered discontinued if the gap between the previous dispensing’s last date of supply and the subsequent refill exceeded 30 days [19]. Sensitivity analysis was performed using a gap of 60 days (instead of 30 days) to define the discontinuation of rifaximin use. The rifaximin dosage was assumed to be 1100 mg per day [18,19]. All the supply from the previous dispensing was used to evaluate the gap.

To assess the adherence and persistence, prescriptions for both rifaximin formulations (200 mg and 550 mg) were considered.

### 2.3. Statistical Analysis

For each patient in cohort 1 and cohort 2, the following variables were assessed at the index date: age group (i.e., 18–64, 65–74 and 75 years and older), sex, cirrhosis complications and any comorbidity. The cirrhosis complications and comorbidities were identified using ICD9-CM codes recorded in all HDRs during the period of 7 years preceding the index date. The following cirrhosis complications were identified: ascites, varices with and without bleeding, portal hypertension, hepatocellular carcinoma (HCC) and jaundice, peritonitis and hepatorenal syndrome. Cardio- and cerebrovascular disease, diabetes, renal disease, psychiatric conditions and cancer (excluding HCC) were grouped as extra-hepatic comorbidities. According to the number of extra-hepatic comorbidities evaluated, patients were classified into three groups: subjects without extra-hepatic comorbidities (a number equal to 0), those with a number equal to 1 and those with a number ≥2.

Data were summarized using percentages for categorial variables and χ^2^ tests were used to compare categorical variables. Survival curves were outlined using the Kaplan–Meier method to verify the time until treatment discontinuation at 12 months. The log-rank test was used to investigate the differences among the age groups curves for the medication persistence outcome.

For our analyses, *p* < 0.05 indicated statistical significance. The analyses were conducted using R statistical software version 4.0.3. and STATA software version 11.

## 3. Results

For cohort 1, a total of 654 patients who had an HE-related hospital admission during the 2019 were recorded. Of those, 106 (16%) subjects died during the hospitalization index; therefore, 544 patients met the inclusion criteria of study cohort 1. The baseline demographic and clinical characteristics are reported in Table 1.

**Figure 2:**
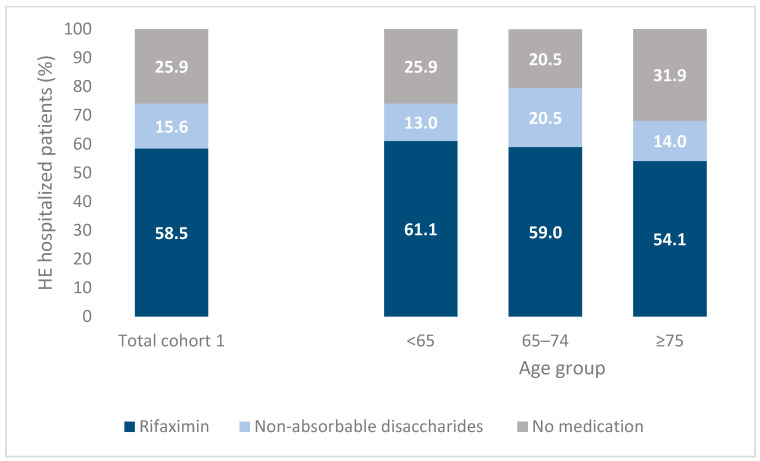
Medication therapy in hepatic encephalopathy (HE) HE hospitalized patients (Cohort 1) within 60 days after discharge: total and stratified by age group.

Of the participants, 61.9% were male and the majority, 39.7%, were younger than 65 years old, followed by 31.4% aged between 65 and 74 and 28.9% older than 75 years. Regarding cirrhosis complications, 52.6% experienced ascites, 39.0% varices and 21.7% portal hypertension. Regarding extra-hepatic comorbidities, diabetes was the most common disease (36.4%) followed by cardiovascular disease (32.9%) and chronic kidney disease (27.2%). Overall, 32.7% had one extra-hepatic comorbidity and 39.2% had two or more. The HE medication therapy for cohort 1 within 60 days post HE discharge is reported in Figure 2. A total of 58.5% patients received a rifaximin prescription, 15.6% non-absorbable disaccharides only, and 25.9% did not receive any HE medications (Figure 2). The proportion of subjects without a HE medication prescription was 31.9% in those aged ≥ 75 years old followed by 25.9% in those younger than 65 years old and 20.5% between 65 and 74 (*p* = 0.063).

**Figure 3:**
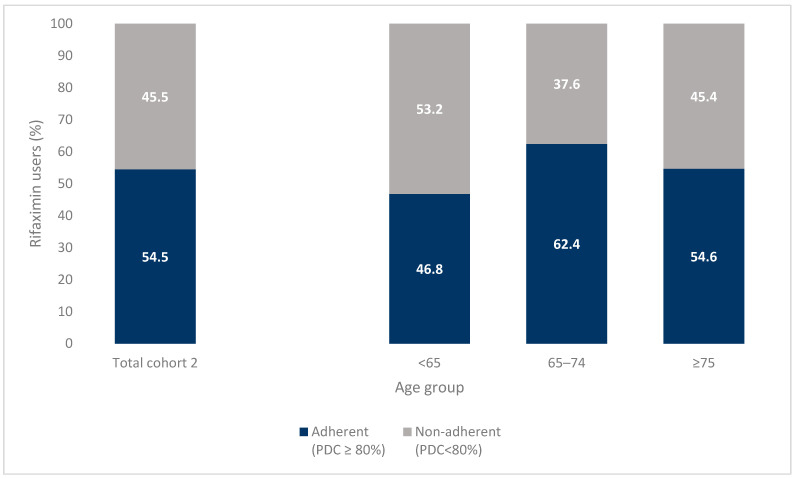
Adherence to rifaximin therapy during the 1-year of the follow-up (Cohort 2): total and stratified by age group.

For cohort 2, a total of 650 incident rifaximin users were selected. Of these, 63.2% were male, 36.8% were younger than 65 years old, 34% were between 65 and 74 years and 29.8% were older than 75 years (Table 1). Of these, 34.3% had ascites, 30.6% varices and 19.7% portal hypertension; 21.2% had one extra-hepatic comorbidity and 28% had two or more (Table 1). The most common extra-hepatic diseases were diabetes (28.5%) and cardiovascular disease (21.2%). During the 1-year follow-up, 54.5% (354 subjects) of the incident rifaximin users had a PDC ≥ 80%, 19.8% a PDC between 50% and 80% and 25.7% a PDC < 50% (Figure 3). Stratifying the cohort by alive and deceased patients during the year of follow-up, the percentage of adherent patients (PDC ≥ 80%) was 60.7% and 51%, respectively.

**Figure 4:**
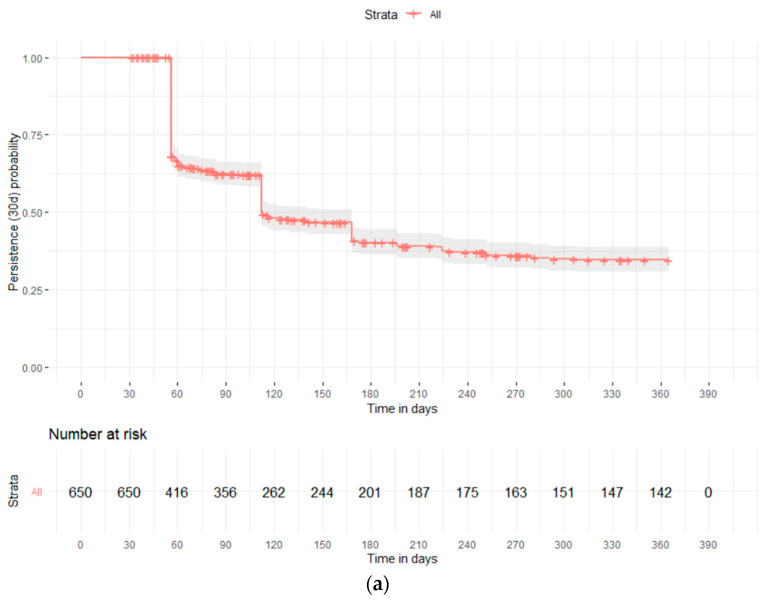

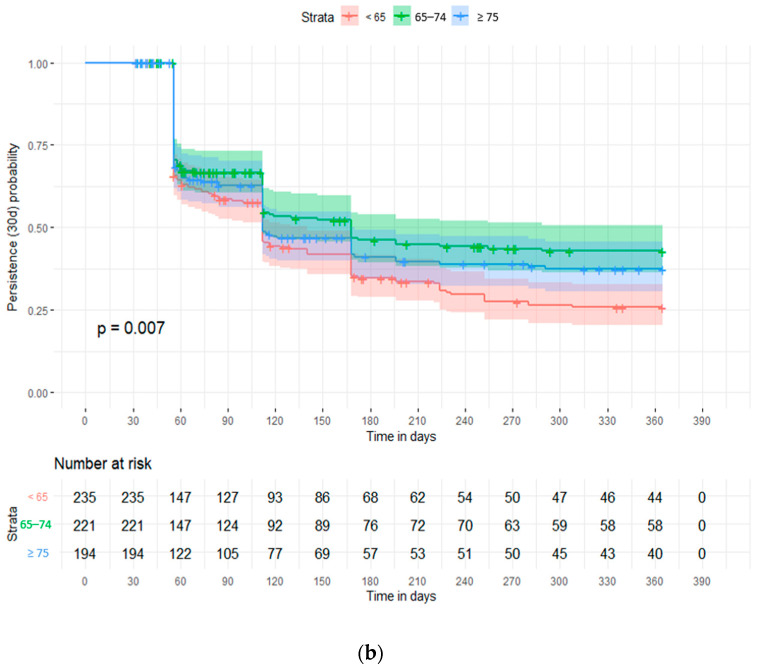
Persistence with rifaximin therapy during the 1 year of follow-up (Cohort 2): total (**a**) and stratified by age group (**b**).

The proportion of adherent users was 46.8% of patients aged <65 years old, 62.4% of those between 64 and 75 years and 54.6% of those older than 75 years (*p* = 0.004). The baseline characteristics of cohort 2, stratified by adherence and nonadherence to the rifaximin therapy, are reported in Table S1. Finally, Figure 4 shows the estimated probability of persistence with rifaximin therapy during the 1-year follow-up. The median survival time of non-persistent patients was 112 days (95% C.I. 112–168). The estimated probability of discontinuing the therapy was 38% at 90 days, 59% at 180 days and 65% at 1 year. Stratifying by age groups, the estimated probability of persistence at 1 year was 26%, 33% and 37% in patients aged <65, 65–74 and ≥75 years old, respectively, with a significant difference among the curves (log-rank test *p* = 0.006). Concerning the rate of rifaximin therapy discontinuation when 60 days was applied as a permissible gap, the estimated probability of non-persistence at 1 year was 47%.

## 4. Discussion

Hepatic encephalopathy represents a major burden on the healthcare system, and reducing the risk of HE recurrence and HE-related hospitalizations is an important part of the treatment paradigm for HE. In our retrospective real-world study, around 58% of patients admitted for an HE event received a rifaximin prescription (with or without non-absorbable disaccharides) within 60 days post-discharge, 15.6% received non-absorbable disaccharides only, and 26% did not receive any medications for HE. Additionally, we found that only 54.5% of patients with HE who initiated treatment with rifaximin were adherent at 1 year and only 35% were persistent beyond a year. Broadly, these findings highlight the need to improve HE therapeutic management in order to reduce the high morbidity and mortality related to HE.

Reduced rates of hospitalization associated with rifaximin treatment have been observed in the prior literature, including clinical trials and real-world studies. A systematic review and meta-analysis that included seven RCTs and enrolled 843 patients with HE found that rifaximin as an add-on to lactulose could provide additional benefits in terms of an increased effective rate and decreased mortality compared to using lactulose alone in patients with HE [24]. In a real-world study considering data spanning from 2014 to 2020 in two US large claims databases, patients had 43–57% lower rates of inpatient days related to HE during rifaximin (with or without lactulose) episodes compared to lactulose episodes [25]. More recently, Stoll et al. reported that only 58% of patients had access to rifaximin at discharge and, in those patients, rifaximin use was associated with a significantly reduced risk of hospitalization and office visits for HE within 12 months of discharge [26]. In total, in our study, 59% of patients were readmitted for any cause during the 1-year follow-up, with HE cited most often as the cause for readmission (Table S3). In line with other findings, we found a high percentage of recurrence within 60 days after the hospital discharge [27]. Notably, 25% of our subgroup of patients was readmitted within 21 days and 50% within 60 days from the discharge index, highlighting the need for proper management immediately after an HE episode [13,14,15,28,29,30]. Not unexpectedly, we also observed that the mortality rate at 1 year was significantly greater in patients with a hospital readmission post-discharge compared with patients not readmitted during this timeframe (57% vs. 47%, respectively; *p* < 0.001) (data not shown). The high mortality rate and high rehospitalization rates highlight the importance of effective HE treatments in order to resolve HE episodes and prevent recurrences [31,32]. Nevertheless, despite current guideline recommendations, we found that 26% of patients discharged for HE did not receive any HE medications, either rifaximin or nonabsorbable disaccharides, within 60 days from the discharge index. The low medication use is concerning and presents opportunities for both physician education regarding the guidelines as well as care-coordination among patients who are discharged from the hospital after an HE episode. Moreover, improving evidence-based medication can also translate into cost savings in this high-risk population. Recently, a meta-analysis of published cost-effectiveness studies of rifaximin in HE, for different lines of treatment and over a range of time horizons, indicated that add-on rifaximin therapy was cost-effective compared with lactulose monotherapy in the second-line setting at 2 years, 5 years, 10 years and lifetime time horizons [33].

Another important aspect to consider is the adherence to rifaximin treatment. In our study, we found that only 54.5% of HE patients who initiated rifaximin treatment exceeded a cut-off point of 80% for the PDC, defined as threshold for good adherence. About 50% of patients discontinued the treatment within the first 6 months. Adherence to rifaximin treatment has been poorly investigated in real life. Volk et al. reported a mean (SD) PDC for treatment during rifaximin episodes equal to 86% (18%) in the Marketscan database and 85% (18%) in the Optum database [25]. Data have indicated that nonadherence to hepatic encephalopathy prophylactic therapies may negatively impact hospital-related outcomes. The authors, taking into account the results from the MarketScan database in a simulated plan of 1 million lives, showed that if payers and physicians ensured proper adherence to rifaximin, the total cost savings would be USD 5.9 million per year. Therefore, effort is required in emphasizing adherence to HE-related medications but also in understanding the reasons for nonadherence. The literature reports that fatigue and confusion and a lack of social support likely have a negative influence on compliance, and complex therapeutic regimens may also contribute to the lack of adherence [34]. HE awareness is often poor in both patients and caregivers, most likely due to insufficient/inadequate provision of information. Providing structured information for the understanding of HE might be relevant to its prevention and its management [35]. Key education points include the effects of medication and potential side-effects, the importance of adherence and early signs of recurrence and appropriate actions.

The findings of this study should be interpreted in light of some limitations. This study relied on ICD9-CM codes recoded on HDRs for establishing the diagnosis of HE; therefore, omission or miscoding may have led to the underestimation of patients admitted for HE. Moreover, as the data source of the study was a single Italian region, the generalizability of our results is limited. A major limitation of the analysis of administrative databases is represented by the absence of single additional clinical information and the severity of the disease. Filling a prescription does not necessarily imply the use of the drug. However, the use of filled prescriptions as a measure of drug exposure eliminates recall bias and improves the accuracy of the information on the drug use [36]. Furthermore, the exact time of discontinuation of therapy was unknown to us. Nonetheless, the results of the study were confirmed by the sensitivity analysis when a different gap was applied to define discontinuation of rifaximin use. 

## 5. Conclusions

This real-world study highlighted that urgent quality improvement is needed to potentially improve the outcomes of HE patients. Rifaximin is recommended by the current guidelines as secondary prophylaxis for the prevention of recurrence of overt hepatic encephalopathy in combination with lactulose. For any patient with cirrhosis and a history of hepatic encephalopathy, prophylactic therapy should be prescribed at hospital discharge to reduce the risk of HE recurrence and hospital readmission. However, effective HE management is reliant on patient adherence to treatment. Therefore, it is essential to improve medication adherence through the implementation of both patient and caregiver education programs to emphasize the long-term value of good compliance.

## Figures and Tables

**Table 1:** Baseline demographic and clinical characteristics of HE hospitalized patients (cohort 1) and incident rifaximin users (cohort 2).

	HE Hospitalized Patients		Incident Rifaximin Users	
	N = 544		N = 650	
	n	%	n	%
**Gender**				
Male	337	61.9	411	63.2
Female	207	38.1	239	36.8
**Age (years)**				
<65	216	39.7	235	36.2
65–74	171	31.4	221	34.0
≥75	157	28.9	194	29.8
**Cirrhosis complication**				
Ascites	286	52.6	223	34.3
Varices	212	39.0	199	30.6
Portal hypertension	118	21.7	128	19.7
Hepatocellular carcinoma	107	19.7	99	15.2
Other *	65	11.9	40	6.2
**Extra-hepatic comorbidities**				
Diabetes	198	36.4	185	28.5
Cardiovascular disease	179	32.9	138	21.2
Chronic kidney disease	148	27.2	93	14.3
Cerebrovascular disease	132	24.3	85	13.1
Cancer	46	8.5	76	11.7
Psychiatric conditions	9	1.7	13	2.0
**Number of extra-hepatic comorbidities**				
0	153	28.1	330	50.8
1	178	32.7	138	21.2
≥2	213	39.2	182	28.0

* Including jaundice, peritonitis and hepatorenal syndrome.

## Data Availability

Restrictions apply to the availability of these data. The readers may contact the authors to access these data.

## Supplementary Materials

The following supporting information can be downloaded at: https://www.mdpi.com/article/10.3390/jcm12134515/s1. Table S1: baseline demographic and clinical characteristics of incident rifaximin users (cohort 2), stratified by adherence; Table S2: baseline demographic and clinical characteristics of incident rifaximin users (cohort 2), stratified by persistence; Table S3: mortality and hospital admission for study cohort 1 during 1 year of follow-up.
