# Supplementary material for: Rifaximin Use, Adherence and Persistence in Patients with Hepatic Encephalopathy: A Real-World Study in the South of Italy
Source: J Clin Med. 2023 Jul 6;12(13):4515. doi: 10.3390/jcm12134515 (PMC10342713; doi:10.3390/jcm12134515)
Supplement: Supplementary file 1 [file jcm-12-04515-s001.zip › jcm-2441753-supplementary.pdf]

Table S1. Baseline demographic and clinical characteristics of incident rifaximin users (Cohort 2), stratified by adherence.

	**Adherents (PDC ≥ 80%) N=354**		**Non-adherents (PDC < 80%) N=296**		**P-value**
	**n**	**%**	**n**	**%**	
**Gender**					
Male	228	64.4	183	61.8	0.496
Female	126	35.6	113	38.2	
**Age (years)**					
<65	110	31.1	125	42.2	
65-74	138	39.0	83	28.0	0.004
≥75	106	29.9	88	29.7	
**Cirrhosis complication**					
Ascites	123	34.7	100	33.8	0.797
Varices	116	32.8	83	28.0	0.193
Portal hypertension	61	17.2	67	22.6	0.084
Hepatocellular carcinoma	57	16.1	42	14.2	0.499
Other*	16	4.5	24	8.1	0.058
**Extra hepatic comorbidities**					
Diabetes	107	30.2	78	26.4	0.276
Cardiovascular disease	90	25.4	48	16.2	0.004
Cerebrovascular disease	55	15.5	38	12.8	0.328
Chronic Kidney Disease	46	13.0	39	13.2	0.946
Cancer	39	11.0	37	12.5	0.558
Psychiatric conditions	5	1.4	8	2.7	0.250
**Number of extra hepatic comorbidities**					
0	167	47.2	163	55.1	
1	79	22.3	59	19.9	0.116
≥2	108	30.5	74	25.0	

* Including jaundice, peritonitis and hepatorenal syndrome.

Table S2. Baseline demographic and clinical characteristics of incident rifaximin users (Cohort 2), stratified by persistence.

	**Persistent N=271**		**Non-persistent N=379**		**P-value**
	**n**	**%**	**n**	**%**	
**Gender**					
Male	172	63.5	239	63.1	0.915
Female	99	36.5	140	36.9	
**Age (years)**					
<65	74	27.3	161	42.5	
65-74	110	40.6	111	29.3	<0.001
≥75	87	32.1	107	28.2	
**Cirrhosis complication**					
Ascites	106	39.1	117	30.9	0.029
Varices	85	31.4	114	30.1	0.726
Portal hypertension	46	17.0	82	21.6	0.141
Hepatocellular carcinoma	48	17.7	51	13.5	0.137
Other*	15	5.5	25	6.6	0.579
**Extra hepatic comorbidities**					
Diabetes	88	32.5	97	25.6	0.055
Cardiovascular disease	66	24.4	72	19.0	0.100
Cerebrovascular disease	41	15.1	52	13.7	0.613
Chronic Kidney Disease	38	14.0	47	12.4	0.546
Cancer	33	12.2	43	11.3	0.745
Psychiatric conditions	4	1.5	9	2.4	0.420
**Number of extra hepatic comorbidities**					
0	128	47.2	202	53.3	
1	57	21.0	81	21.4	0.115
≥2	86	31.8	96	25.3	

* Including jaundice, peritonitis and hepatorenal syndrome.

Table S3. Mortality and hospital admission for the study cohort 1 during 1 year of follow-up.

	Patients with HE-related hospital discharge N=544
**Mortality at 1 year, n (%)**	287 (52.8)
**Hospitalizations for HE, n (%)**	170 (31.2)
**Hospitalizations for any cause, n (%)***	321 (59.0)
**Time to hospitalization***	
Mean±SD	75±75
Percentiles, n	
25	21
50	49
75	103

* Patients re-admitted for any cause within a 1-year post-discharge.
